# Supplementary material for: Biomanufacturing Biotinylated Magnetic Nanomaterial via Construction and Fermentation of Genetically Engineered Magnetotactic Bacteria
Source: Bioengineering (Basel). 2022 Jul 30;9(8):356. doi: 10.3390/bioengineering9080356 (PMC9404834; doi:10.3390/bioengineering9080356)
Supplement: Supplementary file 1 [file bioengineering-09-00356-s001.zip › bioengineering-1781094-supplementary.pdf]

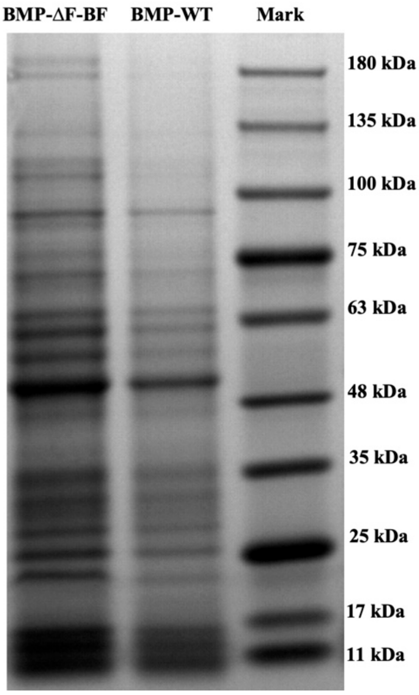

Figure S1. The sodium dodecylsulphate polyacrylamide gel electrophoresis (SDS-PAGE) result of BMP-ΔF-BF and BMP-WT. The pBBR1MCS-2 was a low copy expression vector, so there were no obvious differences between the protein band of BMP-ΔF-BF and BMP-WT.
